# High-frequency electrical stimulation (HFES) Data Lean and Obese Zucker Rat Soleus Muscle: Regulation of p70S6kinase Associated Proteins

**DOI:** 10.1016/j.dib.2017.11.029

**Published:** 2017-11-11

**Authors:** Kevin M. Rice, Anjaiah Katta, Nandini D.P.K. Manne, Ravikumar Arvapalli, Gautam K. Ginjupalli, Miaozong Wu, Shinichi Asano, Eric R. Blough

**Affiliations:** a Center for Diagnostic Nanosystems, Marshall University, Huntington, WV, USA; b Department of Internal Medicine, Joan C. Edwards School of Medicine, Marshall University, Huntington, WV, USA; c Biotechnology Graduate Program West Virginia State University, Institute, WV, USA; d Department of Health and Human Service, School of Kinesiology, Marshall University, Huntington, WV, USA; e Department of Public Heath, Marshall University, Huntington, WV, USA; f College of Health, Science, and Technology, University of Central Missouri, Warrensburg, MO, USA; g School of Education, Health, and Human Performance, Fairmont State University, Fairmont, WV, USA; h Department of Pharmaceutical Sciences and Research, School of Pharmacy, Marshall University, Huntington, WV, USA; i Department of Pharmacology, Physiology and Toxicology, Joan C. Edwards School of Medicine, Marshall University, Huntington, WV, USA

**Keywords:** Diabetes, Skeletal muscle, High-frequency electrical stimulation (HFES), Zucker rat, Soleus, p70s6k

## Abstract

Anaerobic exercise has been advocated as a prescribed treatment for the management of diabetes: however, alterations in exercise-induced signaling remain largely unexplored in the diabetic muscle. Here, we compare the basal and the in situ contraction-induced phosphorylation of the AKT, GSK3beta, mTor, p70s6K, Pten, and Shp2 proteins in the lean and obese (fa/fa) Zucker rat soleus muscle following a single bout of contractile stimuli. This article represents data associated with prior publications from our lab (Katta et al., 2009a, 2009b; Tullgren et al., 1991) [1–3] and concurrent Data in Brief articles (Ginjupalli et al., 2017a, 2017b; Rice et al., 2017a, 2017b) [4–7].

**Specifications Table**

**Table:** 

Subject area	*Biology*
More specific subject area	*Diabetic skeletal muscle response to exercise*
Type of data	*Graph, figure*
How data was acquired	*Immunoblotting*
Data format	*Analyzed*
Experimental factors	*A high-frequency electrical stimulation (HFES) was used to produce 10 sets of 6 contractions over a 22-minute period. Tissues were collected and protein was then isolated from tissue for western blot analysis.*
Experimental features	*Soleus obtained from Lean and Obese male Zucker rats were used in this experiment*
Data source location	*Huntington, WV USA*
Data accessibility	*Data is with this article and is related to articles published and in review*[1], [2], [3], [4], [5], [6], [7]

**Value of the data**

• The data presented in this Brief is vital to understanding the effect of diabetes on skeletal muscle mechanotransduction.
• This data gives insight into how diabetes alters tissue response to stimuli.
• This data provides a more thorough understanding of the mTor pathway involvement in exercise mediated signaling in both diabetic and non-diabetic muscle tissue.

## 1 Data

### 1.1 AKT

**Fig. 1:**
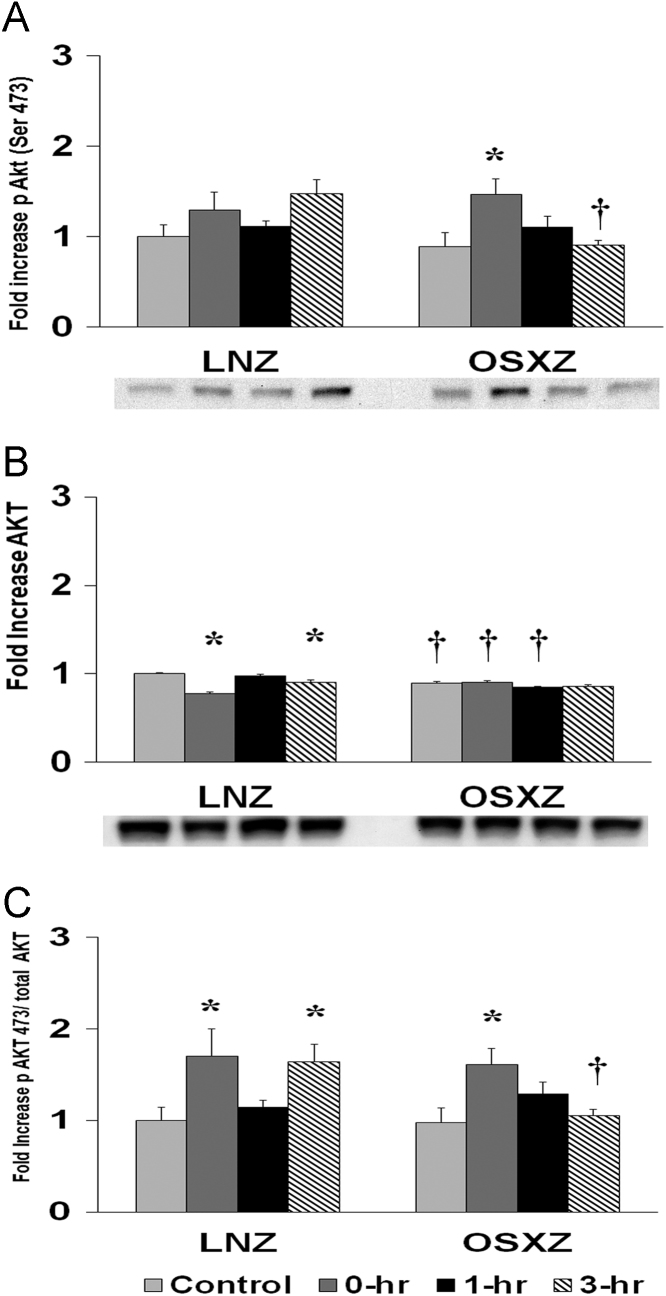
Diabetes alters HFES-induced expression and phosphorylation of Akt rat soleus. The basal (control) and HFES-induced expression of Akt in soleus from non-diabetic lean Zucker (LNZ) and diabetic obese syndrome X Zucker (OSXZ) rats. * Significantly different from HFES soleus within the same group (*p* < 0.05). † Significantly different from corresponding LNZ soleus (*p* < 0.05). *n* = 6/group.

To determine the effect of high-frequency electrical stimulation (HFES) on soleus in diabetic male obese syndrome-X Zucker (OSXZ) diabetic and nondiabetic male normal lean Zucker (LNZ) animals we evaluated the expression of AKT. Soleus basal AKT content was lower (9.8 ± 1.9%, *p* < 0.05) in the OSXZ when compared to LNZ. HFES resulted in a decrease in AKT in the LNZ soleus (22.7 ± 1.9%, 9.8 ± 3.2%, at 0 and 3 hours, *p* < 0.05) when compared to LNZ contralateral control. However, HFES elicited no change in the OSZX soleus when compared to contralateral OXSZ control (Fig. 1).

To determine the effect of HFES on soleus in OSXZ and LNZ animals we evaluated the phosphorylation of AKT at serine 473. Soleus basal phosphorylation of AKT ser 473 demonstrated no difference in the OSXZ when compared to LNZ. HFES elicited not change in phosphorylation of AKT ser 473 in the LNZ soleus when compared to LNZ contralateral control. HFES resulted in an increase in phosphorylation of AKT ser 473 in the OSXZ soleus (58.0 ± 16.7%, at 0 h, *p* < 0.05) when compared to OSXZ contralateral control (Fig. 1).

To determine the effect of HFES on soleus in OSXZ and LNZ animals we evaluated the ratio of phosphorylation of AKT ser 473 to total AKT. Soleus basal phosphorylation of AKT ser 473 to total AKT demonstrated no difference in the OSXZ when compared to LNZ. HFES resulted in an increase in phosphorylation of AKT ser 473 to total AKT in the LNZ soleus (70.1 ± 29.8%, and 64.3 ± 19.1%, at 0 and 3 h, *p* < 0.05) when compared to LNZ contralateral control. HFES resulted in an increase in phosphorylation of AKT ser 473 to total AKT in the OSXZ soleus (63.17% at 0 h, *p* < 0.05) when compared to OSXZ contralateral control (Fig. 1).

### 1.2 GS3K-β

**Fig. 2:**
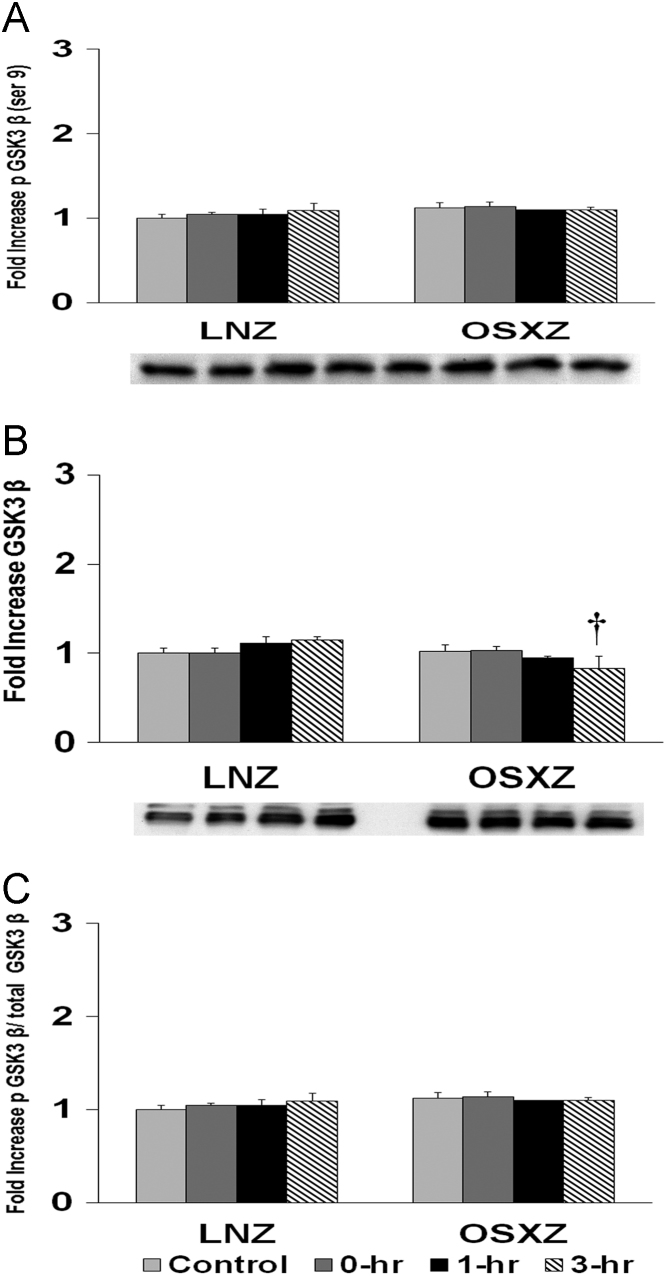
Diabetes alters HFES-induced expression and phosphorylation of GSK3β rat soleus. The basal (control) and HFES-induced expression of GSK3β in soleus from non-diabetic lean Zucker (LNZ) and diabetic obese syndrome X Zucker (OSXZ) rats. ^⁎^ Significantly different from HFES soleus within the same group (*p* < 0.05). † Significantly different from corresponding LNZ soleus (*p* < 0.05). *n* = 6/group.

To determine the effect of HFES on soleus in OSXZ and LNZ animals we evaluated the expression of GS3K-β. Soleus basal GS3K-β content demonstrated no significant change in the OSXZ when compared to LNZ. HFES resulted in no significant change in GS3K-β in the LNZ soleus when compared to LNZ contralateral control. HFES resulted in no significant change in the OSZX soleus when compared to contralateral OXSZ control (Fig. 2).

To determine the effect of HFES on soleus in OSXZ and LNZ animals we evaluated the phosphorylation of GS3K-β at serine 9. Soleus basal phosphorylation of GS3K-β ser 9 demonstrated no significant difference in the OSXZ when compared to LNZ. HFES resulted in no significant change in phosphorylation of GS3K-β ser 9 in the LNZ soleus when compared to LNZ contralateral control. HFES demonstrated no significant change in GS3K-β ser 9 in OSXZ soleus when compared to OSXZ contralateral control (Fig. 2).

To determine the effect of HFES on soleus in OSXZ and LNZ animals we evaluated the phosphorylation of GS3K-β ser 9 to total GS3K-β. Soleus basal phosphorylation of GS3K-β ser 9 to total GS3K-β was not significantly different in the OSXZ when compared to LNZ. HFES elicited no significant change in phosphorylation of GS3K-β ser 9 to total GS3K-β in the LNZ soleus when compared to LNZ contralateral control. HFES elicited no significant change in phosphorylation of GS3K-β ser 9 to total GS3K-β in the OSXZ soleus when compared to OSXZ contralateral control (Fig. 2).

### 1.3 mTor

**Fig. 3:**
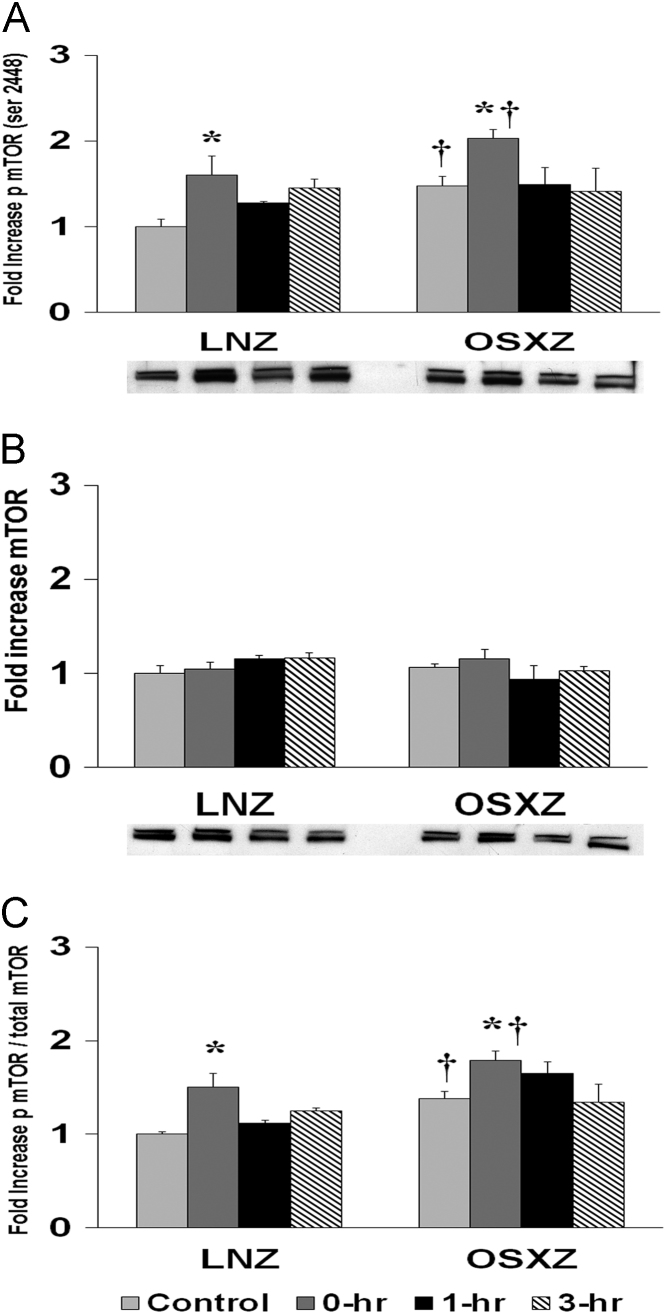
Diabetes alters HFES-induced expression and phosphorylation of mTor rat soleus. The basal (control) and HFES-induced expression of mTor in soleus from non-diabetic lean Zucker (LNZ) and diabetic obese syndrome X Zucker (OSXZ) rats. ^⁎^ Significantly different from HFES soleus within the same group (*p* < 0.05). † Significantly different from corresponding LNZ soleus (*p* < 0.05). *n* = 6/group.

To determine the effect of HFES on soleus in OSXZ and LNZ animals we evaluated the expression of mTor. Soleus basal mTor content demonstrated no significant difference in the OSXZ when compared to LNZ. HFES resulted in no significant change in mTor in the LNZ soleus when compared to LNZ contralateral control. HFES did not produce a significant change in mTor in the OSXZ soleus when compared to contralateral OXSZ control (Fig. 3).

To determine the effect of HFES on soleus in OSXZ and LNZ animals we evaluated the phosphorylation of mTor at serine 2448. Soleus basal phosphorylation of mTor ser 2448 was higher (47.4 ± 11.6%, *p* < 0.05) in the OSXZ when compared to LNZ. HFES resulted in an increase in phosphorylation of mTor at serine 2448 in the LNZ soleus (60.1 ± 22.4%, at 0 h, *p* < 0.05) when compared to LNZ contralateral control. HFES resulted in an increase in phosphorylation of mTor at serine 2448 in the OSXZ soleus (55.4 ± 10.6%, at 0 h, *p* < 0.05) when compared to OSXZ contralateral control (Fig. 3).

To determine the effect of HFES on soleus in diabetic male OSXZ and LNZ animals we evaluated the phosphorylation of mTor at serine 2448 to total mTor. Soleus basal phosphorylation of mTor ser 2448 to total mTor was higher (38.3 ± 7.3%, *p* < 0.05) in the OSXZ when compared to LNZ. HFES resulted in an increase (50.62 ± 14.6%, at 0 hours, *p* < 0.05) in phosphorylation of mTor ser 2448 to total mTor in the LNZ soleus when compared to LNZ contralateral control. HFES resulted in an increase in phosphorylation of mTor ser 2448 to total mTor in the OSXZ SOLEUS (41.0, at 0 h, *p* < 0.05) when compared to OSXZ contralateral control (Fig. 3).

### 1.4 P70s6k

**Fig. 4:**
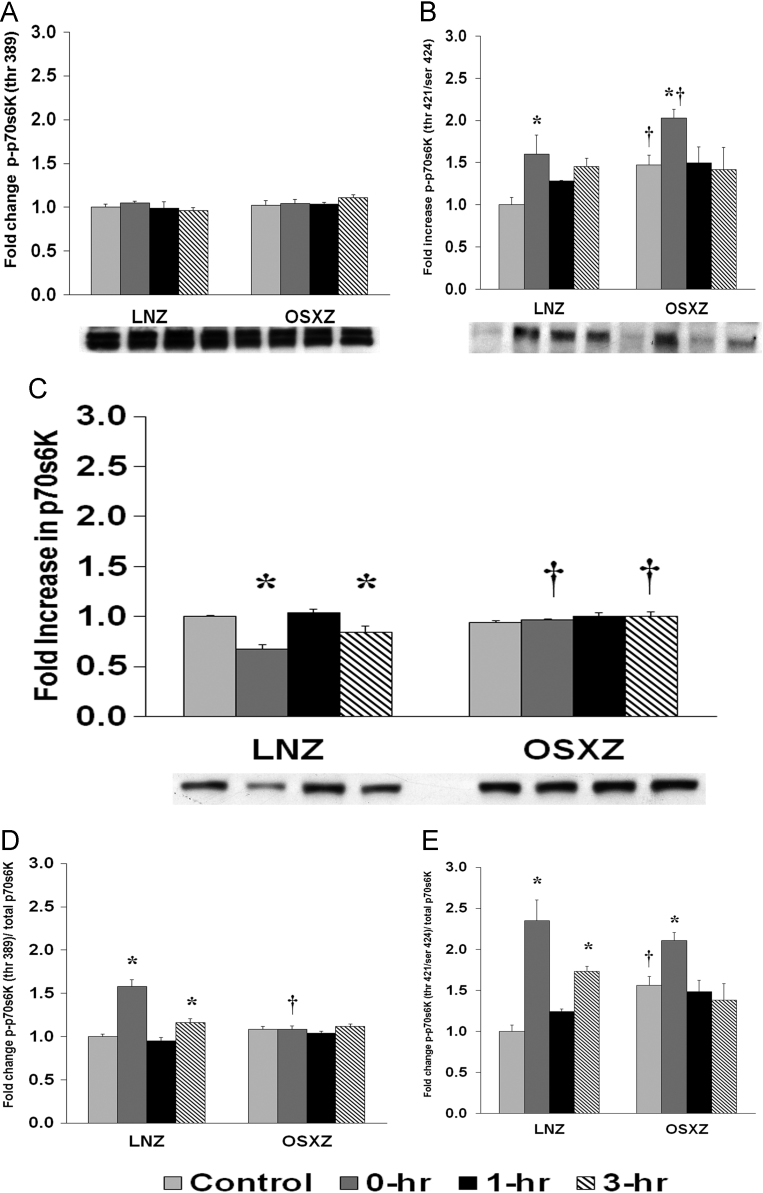
Diabetes alters HFES-induced expression and phosphorylation of p70s6K rat soleus. The basal (control) and HFES-induced expression of p70s6K in soleus from non-diabetic lean Zucker (LNZ) and diabetic obese syndrome X Zucker (OSXZ) rats. ^⁎^ Significantly different from HFES soleus within the same group (*p* < 0.05). † Significantly different from corresponding LNZ soleus (*p* < 0.05). **n** = 6/group.

To determine the effect of HFES on soleus in OSXZ and LNZ animals we evaluated the expression of p70s6k. Soleus basal p70s6k was not significantly different in the OSXZ when compared to LNZ. HFES resulted in a decrease (32.4 ± 4.7%, and 15.5 ± 6.2%, at 0 and 3 hours, *p* < 0.05) in p70s6k in the LNZ SOLEUS when compared to LNZ contralateral control. HFES elicited no change in the OSZX SOLEUS when compared to contralateral OXSZ control (Fig. 4).

To determine the effect of HFES on soleus from OSXZ and LNZ animals we evaluated the phosphorylation of p70s6k at threonine 389 (thr 389) and threonine 421 serine 424 (thr 421/ser 424). Soleus basal phosphorylation of p70s6k thr 389 demonstrated no change in the OSXZ when compared to LNZ. HFES elicited no change in phosphorylation of p70s6k (thr 389) in the LNZ soleus when compared to LNZ contralateral control. HFES elicited not change in OSXZ soleus when compared to OSXZ contralateral control. Soleus basal phosphorylation of p70s6k thr 421/ser 424 was higher (47.4 ± 11.6%, *p* < 0.05) in the OSXZ when compared to LNZ. HFES resulted in an increase (60.1 ± 22.4%, at 0 h, *p* < 0.05) in phosphorylation of p70s6k thr 421/ser 424 in the LNZ soleus when compared to LNZ contralateral control. HFES resulted in an increase in phosphorylation of p70s6k thr 421/ser 424 in the OSXZ soleus (55.4 ± 10.6%, at 0 hours, *p* < 0.05) when compared to OSXZ contralateral control (Fig. 4).

To determine the effect of HFES on soleus in OSXZ and LNZ animals we evaluated the phosphorylation of p70s6k thr 389 and p70s6k thr 421/ser 424 to total p70s6k. Soleus basal phosphorylation of p70s6k thr 389 to total p70s6k was not significantly different in the OSXZ when compared to LNZ. HFES resulted in an increase in phosphorylation of p70s6k thr 389 to total p70s6k in the LNZ soleus (57.9 ± 7.8% and 15.9 ± 5.0%, at 0 and 3 h, *p* < 0.05) when compared to LNZ contralateral control. HFES did not elicit a change in phosphorylation of p70s6k thr 389 to total p70s6k in the OSXZ soleus when compared to OSXZ contralateral control. Soleus basal phosphorylation of p70s6k thr 421/ser 424 to total p70s6k was higher (56.4 ± 10.4%, *p* < 0.05) in the OSXZ when compared to LNZ. HFES resulted in an increase (134.9 ± 25.2% and 72.9 ± 6.5%, at 0 and 3 hours, *p* < 0.05) in phosphorylation of p70s6k thr 421/ser 424 to total p70s6k in the LNZ soleus when compared to LNZ contralateral control. HFES resulted in an increase in phosphorylation of p70s6k thr 421/ser 424 to total p70s6k in the OSXZ soleus (54.2 ± 9.7%, at 0 h, *p* < 0.05) when compared to OSXZ contralateral control (Fig. 4).

### 1.5 PTEN

**Fig. 5:**
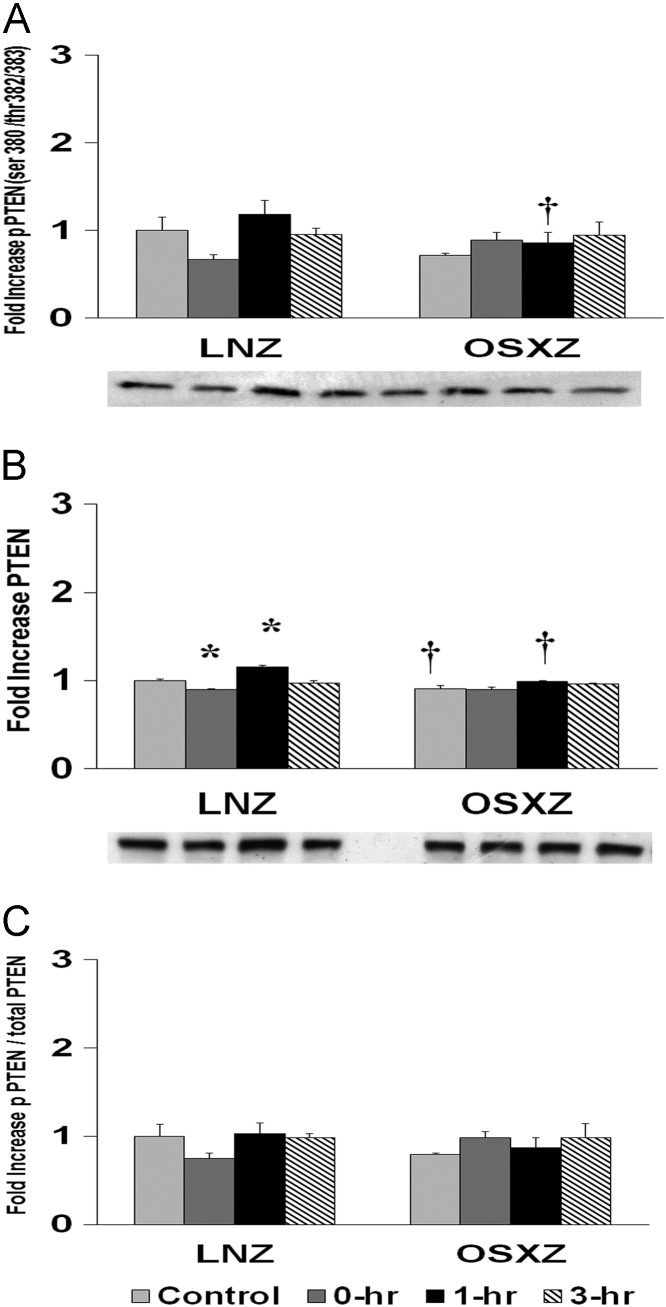
Diabetes alters HFES-induced expression and phosphorylation of PTEN rat soleus. The basal (control) and HFES-induced expression of PTEN in soleus from non-diabetic lean Zucker (LNZ) and diabetic obese syndrome X Zucker (OSXZ) rats. ^⁎^ Significantly different from HFES soleus within the same group (*p* < 0.05). † Significantly different from corresponding LNZ soleus (*p* < 0.05). *n* = 6/group.

To determine the effect of HFES on soleus in OSXZ and LNZ animals we evaluated the expression of PTEN. Soleus basal PTEN was significant lower (9.5 ± 4.3%, *p* < 0.05) in the OSXZ when compared to LNZ. HFES resulted in a decrease (10.4 ± 0.8%, at 0 h, *p* < 0.05) and an increase (15.0 ± 2.4%, at 3 h, *p* < 0.05) in PTEN in the LNZ soleus when compared to LNZ contralateral control. HFES did not elicit a change in PTEN in the OSZX SOLEUS when compared to contralateral OXSZ control (Fig. 5).

To determine the effect of HFES on soleus in OSXZ and LNZ animals we evaluated the phosphorylation of PTEN at serine 380, threonine 382 and threonine 383 (PTEN ser 380/thr 382/383). Soleus basal phosphorylation of PTEN ser 380/thr 382/383 was not significantly different in the OSXZ when compared to LNZ. HFES did not elicit a change in phosphorylation of PTEN ser 380/thr 382/383 in the LNZ soleus when compared to LNZ contralateral control. HFES did not elicit a change in phosphorylation of PTEN ser 380/thr 382/383 in the OSXZ soleus in OSXZ soleus when compared to OSXZ contralateral control (Fig. 5).

To determine the effect of HFES on soleus in OSXZ and LNZ animals we evaluated the phosphorylation of PTEN ser 380/thr 382/383 to total PTEN. Soleus basal phosphorylation of PTEN ser 380/thr 382/383 to total PTEN was no significantly different in the OSXZ when compared to LNZ. HFES did not elicit a change in phosphorylation of PTEN ser 380/thr 382/383 to total PTEN in the LNZ soleus when compared to LNZ contralateral control. HFES did not elicit a change in phosphorylation of PTEN ser 380/thr 382/383 to total PTEN in the OSXZ soleus when compared to OSXZ contralateral control (Fig. 5).

### 1.6 SHP2

**Fig. 6:**
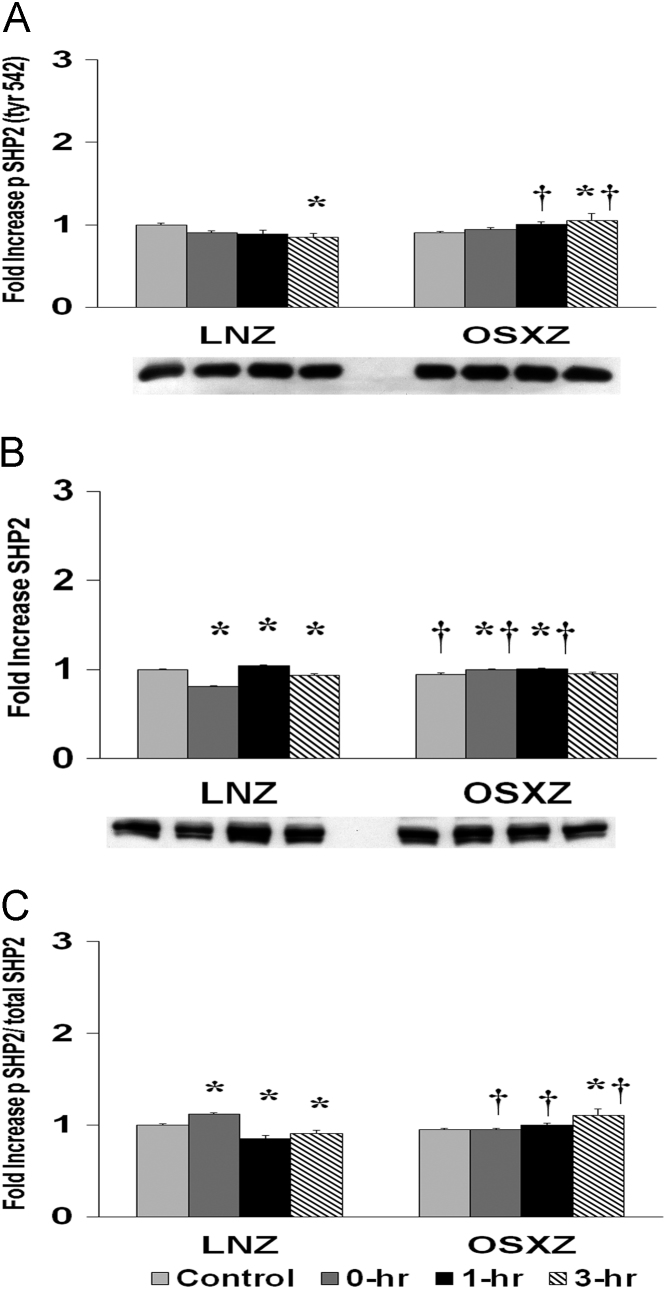
Diabetes alters HFES-induced expression and phosphorylation of SHP2 rat soleus. The basal (control) and HFES-induced expression of p42/p44 in soleus from non-diabetic lean Zucker (LNZ) and diabetic obese syndrome X Zucker (OSXZ) rats. ^⁎^ Significantly different from HFES soleus within the same group (*p* < 0.05). † Significantly different from corresponding LNZ soleus (*p* < 0.05). *n* = 6/group.

To determine the effect of HFES on soleus in OSXZ and LNZ animals we evaluated the expression of SHP23. Soleus basal SHP2 content was lower (5.2 ± 1.5%, *p* < 0.05) in the OSXZ when compared to LNZ. HFES resulted in a decrease (19.5 ± 1.2% and 6.8 ± 2.0%, at 0 and 3 h, *p* < 0.05) and an increase (4.4 ± 1.1%, at 1 h, *p* < 0.05) in SHP2 in the LNZ soleus when compared to LNZ contralateral control. HFES resulted in an increase (4.6 ± 1.1% and 5.6 ± 1.2%, at 0 and 1 h, *p* < 0.05) in SHP2 in the OSZX soleus when compared to contralateral OXSZ control (Fig. 6).

To determine the effect of HFES on soleus in OSXZ and LNZ animals we evaluated the phosphorylation of SHP2 at tyrosine 542. Soleus basal phosphorylation of SHP2 tyr 542 was not significantly different in the OSXZ when compared to LNZ. HFES resulted in a decrease (15.5 ± 5.2%, at 3 h, *p* < 0.05) in phosphorylation of SHP2 tyr 542 in the LNZ soleus when compared to LNZ contralateral control. HFES resulted in an increase in phosphorylation of SHP2 tyr 542 in the OSXZ soleus (15.3 ± 8.3, at 3 h, *p* < 0.05) when compared to OSXZ contralateral control (Fig. 6).

To determine the effect of HFES on soleus in OSXZ and LNZ animals we evaluated the phosphorylation of SHP2 tyr 542 to total SHP2. Soleus basal phosphorylation of SHP2 tyr 542 to total SHP2 demonstrated no significant difference in the OSXZ when compared to LNZ. HFES resulted in an increase (11.8 ± 1.6%, at 0 h, *p* < 0.05) and a decrease (14.7 ± 3.1% and 9.5 ± 3.8%, at 1 and 3 h, *p* < 0.05) in phosphorylation of SHP2 tyr 542 to total SHP2 in the LNZ soleus when compared to LNZ contralateral control. HFES resulted in an increase in phosphorylation of SHP2 tyr 542 to total SHP2 in the OSXZ soleus (15.0% ± 7.3%, at 3 h, *p* < 0.05) when compared to OSXZ contralateral control (Fig. 6).

## 2 Experimental design, materials and methods

### 2.1 Animals

All procedures were conducted in strict accordance with the Council of the American Physiological Society and the Animal Use Review Board of Marshall University. Young (10 week, *n* = 12) male lean Zucker (non-diabetic) (LNZ) and young (10 week, *n* = 12) male obese syndrome-X Zucker (diabetic) (OSXZ) rats were obtained from the Charles River Laboratories and barrier housed one per cage in an AAALAC approved vivarium. Housing conditions consisted of a 12H: 12H dark-light cycle and the temperature was maintained at 22 ± 2 °C. Animals were provided food and water *ad libitum*. Rats were allowed to recover from shipment for at least two weeks before the commencement of experimentation during which time the animals were carefully observed and weighed weekly.

### 2.2 Materials

Anti-p70S6k (#9202), Akt (#9272), mTOR (#2972), glycogen synthase kinase-3β (GSK-3β) (#9332), PTEN (#9552), Thr389 (#9206) and Ser 421 / Thr 424 (#9204) phosphorylated p70S6K, Thr308 (#9275) and Ser473 (#9271) phosphorylated Akt, Ser2448 phosphorylated mTOR (#2971), Ser 9 phosphorylated GSK-3β (#9336), Ser 380/Thr 382/383 phosphorylated PTEN (#9554), SHP-2 (#3752), p-SHP-2 (Tyr 542) (cat #3751), Mouse IgG, and Rabbit IgG antibodies were purchased from Cell Signaling Technology (Beverly, MA). Enhanced chemiluminescence (ECL) western blotting detection reagent was from Amersham Biosciences (Piscataway, NJ). Precast 10% and 15% SDS-PAGE gels were purchased from Lonza (Rockland, ME). Enhanced chemiluminescence (ECL) western blotting detection reagent was purchased from Amersham Biosciences (Piscataway, NJ). Restore western blot stripping buffer was obtained from Thermo scientific (Rockford, IL). All other chemicals were from Sigma (St. Louis, MO).

### 2.3 Contractile stimulation of skeletal muscles

The high-frequency electrical stimulation (HFES) model has been previously described [8], [9]. The HFES model used in the present study produced 10 sets of 6 contractions with an overall protocol time of 22 min. Animals were killed by a lethal dose of pentobarbital sodium at baseline, immediately following, 1 h or 3 h (*n* = 6 normal, *n* = 6 diabetic for 0, 1, and 3 h) after HFES. Once excised, muscles were blotted dry, trimmed of visible fat and tendon projections, weighed, immediately frozen in liquid nitrogen, and stored at − 80 °C.

### 2.4 Immunoblot analysis

Samples were prepared and immunoblotting performed as described by Rice et al. [1], [2], [3], [4], [5], [6], [7].

### 2.5 Data analysis

Data were analyzed using Sigma Stat 12.0 statistical software and the results are presented as mean ± SEM. Two-way ANOVA followed by the Student-Newman-Keuls post-hoc test was conducted to determine differences between groups. The level of significance accepted *a priori* was < 0.05.
